# Antibiotic‐associated suspected adverse drug reactions among hospitalized patients in Uganda: a prospective cohort study

**DOI:** 10.1002/prp2.298

**Published:** 2017-02-17

**Authors:** Ronald Kiguba, Charles Karamagi, Sheila M. Bird

**Affiliations:** ^1^ Department of Pharmacology and TherapeuticsMakerere University College of Health SciencesKampalaUganda; ^2^ Clinical Epidemiology UnitMakerere University College of Health SciencesKampalaUganda; ^3^ Medical Research Council Biostatistics UnitCambridgeUnited Kingdom

**Keywords:** Adverse drug reaction, antibiotics, defined daily dose, rare adverse drug reaction, serious adverse drug reaction, signal, Uganda

## Abstract

We sought to determine the prevalence at admission and incidence during hospitalization of antibiotic‐associated suspected adverse drug reactions (aa‐ADRs) among Ugandan inpatients; and to characterize these aa‐ADRs. We conducted a prospective cohort study of 762 consented adults admitted on medical and gynecological wards of the 1790‐bed Mulago National Referral Hospital. Thirty percent were known HIV‐seropositive (232/762). Nineteen percent (148/762; 95% CI: 17–22%) of inpatients experienced at least one aa‐ADR. At hospital admission, 6% (45/762; 95% CI: 4–8%) of patients had at least one aa‐ADR; and 15% (45/300; 11–20%) of those who had received antibiotics in the 4‐weeks preadmission. Twenty‐four (53%) of these 45 patients had serious aa‐ADRs. The incidence of aa‐ADRs was 19% (117/629; 95% CI: 16–22%) of patients who received antibiotics [community‐acquired: 9% (27/300; 95% CI: 6–13%); hospital‐acquired: 16% (94/603; 95% CI: 13–19%)]: 39 (33%) of 117 patients had serious aa‐ADRs. Of 269 aa‐ADRs, 115 (43%) were community‐acquired, 66 (25%) probable/definite, 171 (64%) preventable, 86 (32%) serious, and 24 (9%) rare. Ceftriaxone was the most frequently implicated for serious hospital‐acquired aa‐ADRs. Cotrimoxazole, isoniazid, rifampicin, ethambutol, and pyrazinamide were the most frequently linked to serious community‐acquired aa‐ADRs. Fatal jaundice (isoniazid), life‐threatening difficulty in breathing with shortness of breath (rifampicin) and disabling itchy skin rash with numbness of lower swollen legs (ethambutol, isoniazid) were observed. Pharmaceutical quality testing of implicated antibiotics could be worthwhile. Periodic on‐ward collection and analysis of antibiotic‐safety‐data standardized by consumption is an efficient method of tracking antibiotics with 1%‐risk for serious aa‐ADRs.

## Introduction

Antibiotics rank among the most widely prescribed medications globally (WHO, 2004; Shehab et al. 2008; Adriaenssens et al. 2011). Widespread antibiotic use predisposes patients to antibiotic‐associated adverse drug reactions (aa‐ADRs), including serious aa‐ADRs (WHO‐UMC, 2000). Antibiotics contributed to 19% of emergency department visits for suspected ADRs in the United States (US) between 2004 and 2006 (Shehab et al. 2008), 8% of ADRs linked to hospital admissions in Greece in 2005 (Alexopoulou et al. 2008), 6% in Spain between 2001 and 2006 (Carrasco‐Garrido et al. 2010), 5% in The Netherlands in 2003 (van der Hooft et al. 2008), and 11% in India between 2002 and 2009 (Sonal et al. 2011); and are responsible for a considerable proportion of hospital‐acquired ADRs in the US (10%) (Weiss et al. 2011) and South Africa (22%) (Mehta et al. 2008). Allergic reactions constitute 79% of aa‐ADRs at US emergency departments (Shehab et al. 2008).

Developing countries contributed 76% of the global rise in antibiotic use between 2000 and 2010 (Van Boeckel et al. 2014), which increases their risk of aa‐ADRs. Cotrimoxazole use is standard‐of‐care for prophylaxis against opportunistic infections among HIV/AIDS patients in resource‐limited settings (WHO, 2006). Thus, the high burden of HIV/AIDS in sub‐Saharan Africa (SSA) implies increased risk of cotrimoxazole‐linked ADRs (Mouton et al. 2015). Little is known about the frequency (Kiguba et al. 2015) and characteristics of aa‐ADRs among inpatients in SSA (Mehta et al. 2008), particularly in Uganda. Moreover, it is a challenge to ascertain aa‐ADR causality in our hospital setting where a large proportion of inpatients have comorbidities and/or concurrently receive multiple medicines.

Four‐fifths of inpatients at a tertiary care public health facility in Uganda consume at least one antibiotic during hospitalization, whereas two‐fifths use at least one antibiotic during the 4‐weeks preadmission (Kiguba et al. 2016a). Patients can receive parenteral antibiotics at private clinics in the community prior to their subsequent referral to tertiary care public health facilities.

The reported antibiotic prescribing patterns in our hospital setting excluded antituberculous drugs and focused on commonly used antibacterial agents (Kiguba et al. 2016a), whose prescription is not restricted to specialists, to identify key areas for antibiotic stewardship. For the benefit of antituberculous drugs pharmacovigilance in Uganda, this paper includes data on the aa‐ADRs of antituberculous drugs. We sought to determine the prevalence at hospital admission and the incidence during hospital stay of aa‐ADRs among Ugandan inpatients: their seriousness, rarity, preventability, causality, and severity.

## Materials and Methods

### Study design and setting

This prospective cohort study (Kiguba et al. 2016a) was conducted among hospitalized adult patients (≥18 years) at the Mulago National Referral Hospital (1790 beds; more than 140,000 inpatients annually). Briefly, three medical wards were studied: Infectious Diseases and Gastrointestinal Illnesses (IDGI); Haematology, Neurology and Endocrinology (HNE); and Cardiovascular, Pulmonology and Nephrology (CPN); and one Gynecology ward (GYN). IDGI and CPN each admitted 10–15 patients/day and HNE received 5–10 patients/day; therefore 25–40 admissions/day on medical wards. However, GYN had 20–25 admissions/day.

Patients gave written informed consent.

### Data collection

From October to November 2013, we undertook a pilot study on the four wards to assess practicability and to pretest the study instruments. The main study (reported here) was implemented in December 2013 to April 2014. Four trained ward‐teams, each having a medical doctor, pharmacist and degree‐nurse, were to recruit and follow‐up inpatients using a systematic random sampling procedure: three new admissions daily on long‐stay wards (HNE/CPN) and six on short‐stay wards (IDGI/GYN). Ward‐teams randomly selected the first study patient from the first two (IDGI), three (HNE), and four (CPN/GYN) new admissions; and subsequently aimed at recruiting every second, third, and fourth admission, respectively (Kiguba et al. 2016a).

Baseline patient assessment captured relevant data on demographics, clinical conditions including aa‐ADRs, and antibiotic medications used. Subsequently, daily assessments were conducted until discharge, transfer, death, or loss to follow‐up. Ward‐teams collected data from 8.00am to 6.00 pm from Monday to Friday and from10.00am to 6.00 pm on weekends and public holidays (Kiguba et al. 2016a).

### Data management

The data were double‐entered into a database using Epidata 3.1 software (Odense, Denmark) with check programs to limit out‐of‐range data entry errors. Where data discrepancies occurred, the original case report form was cross‐checked and corrections were made.

### Identification of aa‐ADRs

We defined aa‐ADRs according to the WHO definition (WHO‐UMC, 2011). Clinical examination was the major approach used to identify aa‐ADRs due to limitations in timely availability of laboratory investigation results (Kiguba et al. 2016b). To increase the probability to detect aa‐ADRs, patients were screened using an ADR trigger tool (Rozich et al. 2003). To assess causality, a suspected aa‐ADR was assigned to a Naranjo ADR probability category based on a total score obtained from 10 weighted questions. These questions assessed the temporal association between suspected drug and adverse reaction, alternative cause(s) of the reaction, plasma drug levels (if available), dose–response relationships and previous patient experience with the drug. Suspected aa‐ADRs with Naranjo score of 0 were *doubtful,* 1–4 *possible*, 5–8 *probable*, and ≥9 *definite* (Naranjo et al. 1981). Thus, coding an adverse event as “aa‐ADR” required at least *possible* grading on the Naranjo scale. Operationally, an *aa‐ADR* was any undesirable medical occurrence that developed after the administration of an antibiotic and for which there was, at least, *possible* causality between the antibiotic and the medical occurrence. Consensus agreement on aa‐ADR causality was reached in a committee headed by the ward‐based study physician and senior clinical pharmacist (RK). This team approach reflected the routine on‐ward approach whereby nurses, medical doctors, and clinical pharmacists brainstorm on patients' clinical problems before making clinical decisions (Kiguba et al. 2016a). Community‐acquired aa‐ADRs were defined as ADRs linked to preadmission use of antibiotics. Some community‐acquired aa‐ADRs manifested preadmission, whereas others occurred after hospital admission. Hospital‐acquired aa‐ADRs were those linked to hospital‐initiated antibiotics used during the current hospitalization.

Preventability, severity (grade or intensity), and seriousness (incapacitating or life‐threatening) of aa‐ADRs were also determined by consensus as described above. Preventability was assessed using the modified Schumock and Thornton Preventability Scale (Schumock and Thornton 1992; Lau et al. 2003), whereas severity was evaluated using the Division of AIDS Table for Grading the Severity of Adult and Paediatric Adverse Events (Division of AIDS (DAIDS), 2004) and seriousness using the WHO Uppsala Monitoring Centre (UMC) criteria (WHO‐UMC, 2000). Rarity of an aa‐ADR (occurrence in <0.1% of medication users) (WHO‐UMC, 2011), was assessed by RK using the British National Formulary (BNF) (British National Formulary, 2014) as the principal reference.

### Statistical analysis

We determined the prevalence of aa‐ADRs at hospital admission, and the incidence of aa‐ADRs during hospitalization. Numerators for prevalence and incidence were the number of patients who had experienced preadmission aa‐ADRs and new cases of in‐hospital aa‐ADRs, respectively, whereas the denominator was the number of study patients who received antibiotics (both incidence and prevalence) or total number of patients in the cohort (prevalence only).

We also computed the incidence of hospital‐acquired aa‐ADRs per 100 defined daily doses (DDDs) (Anatomical Therapeutic Chemical/Defined Daily Dose (ATC/DDD) Index, 2015; Hamad et al. 2013) of each implicated antibiotic administered during the current hospitalization.

We assessed aa‐ADRs as community‐acquired or hospital‐acquired; and for causality, preventability, severity, seriousness, and rarity.

All statistical analyses were conducted using Stata 12.0 (StataCorp, 2011).

### Ethical clearance

Ethical approval for the study was obtained from the School of Medicine Research and Ethics Committee, Makerere University College of Health Sciences (REC REF No. 2011–113), the Mulago Hospital Research and Ethics Committee (MREC 253), and the Uganda National Council for Science and Technology (HS 1151).

## Results

### Study population

Of 762 inpatients included in the study, 70% (534/762: 191 in gynecology, 343 in the medical wards) were female. Median age of inpatients was 30 years [interquartile range (IQR) of 24–42] and median length of hospital stay was 4 days (IQR: 3–6). Thirty percent were known HIV‐seropositive [(232/762): 38% (215/571) being on the medical wards and 9% (17/191) on gynecology], see Table 1.

**Table 1:** Demographic and clinical characteristics of 762 hospitalized patients, Uganda, 2014

Characteristic	Number of patients (*n* = 762)^a^	Patients with community‐acquired antibiotic‐associated. ADRs at admission	Patients who developed new antibiotic‐associated. ADRs while in hospital^b^	
			Community‐acquired	Hospital‐acquired
Age, years [median, interquartile range (IQR)]	30 (24–42)	33 (27–42)	33 (26–40)	30 (25–39)
Number of administered medicines (median, IQR)	7 (5–10)	10 (8–12)	11 (7–14)	9 (7–12)
Length of hospital stay, days (median, IQR)	4 (3–6)	4 (3–7)	7 (5–9)	6 (5–9)
Characteristic	Number of patients (*n* = 762), % col^a^	Patients with community‐acquired antibiotic‐associated ADRs at admission (% Prevalence)	Patients who developed new antibiotic‐associated ADRs while in hospital (% Incidence)^b^	
			Community‐acquired	Hospital‐acquired
Gender				
Male	228 [30]	20 (9)	9 (4)	30 (13)
Female	534 [70]	25 (4)	14 (3)	64 (12)
Ward‐type				
Medical wards	571 [75]	40 (7)	22 (4)	68 (12)
Infectious diseases & gastrointestinal illnesses	320 [42]	19 (6)	5 (2)	31 (10)
Hematology, neurology & endocrinology	117 [15]	0 (0)	2 (2)	9 (8)
Cardiovascular, pulmonology & nephrology	134 [18]	21 (16)	19 (14)	28 (21)
Gynecology ward	191 [25]	5 (3)	1 (1)	26 (14)
HIV status by ward‐type				
Positive	232 [30]	38 (16)	18 (8)	34 (15)
Medical wards	215 [38]	37 (17)	18 (8)	29 (13)
Gynecology ward	17 [9]	1 (6)	0 (0)	5 (29)
Negative or unknown	530 [70]	7 (1)	9 (2)	60 (11)
Medical wards	356 [62]	3 (1)	8 (2)	39 (11)
Gynecology ward	174 [91]	4 (2)	1 (1)	21 (12)

a Of 762 patients, 300 had received antibiotics preadmission, 603 initiated antibiotics during hospital stay, and 629 received antibiotics either preadmission or during hospital stay; square brackets [] represent column percentages; round brackets () represent row percentages.

b Four patients experienced incident community‐acquired and hospital‐acquired antibiotic‐associated ADRs during the current hospitalization.

### Extent of aa‐ADRs

Overall, 42% (320/762; 95% CI: 38–46%) of patients experienced at least one suspected ADR attributable to any medication class: 46% (148/320; 95% CI: 41–52%) of these patients, or 19% (148/762; 95% CI: 17–22%) of all patients, experienced at least one aa‐ADR, see Table 2. Fifty‐eight percent (86/148) of the patients with aa‐ADRs encountered hospital‐acquired aa‐ADRs only, 37% (54/148) had community‐acquired aa‐ADRs only and 5% (8/148) both community‐acquired (incident and prevalent) and hospital‐acquired aa‐ADRs.

**Table 2:** Extent of antibiotic‐associated suspected ADRs at patient‐level among 762 inpatients, Uganda, 2014

Characteristic at patient‐level	Number of suspected ADRs, *n*		
	HIV+	HIV‐ or Unknown	Total
Suspected ADRs attributed to any medication class	136	184	320
Antibiotic‐associated suspected ADRs	75	73	148
Incident antibiotic‐associated suspected ADRs during hospital stay	51	66	117
Serious incident in‐hospital suspected ADRs	23	16	39
Prevalent antibiotic‐associated suspected ADRs at admission	38	7	45
Serious prevalent suspected ADRs at admission	23	1	24
Community‐acquired antibiotic‐associated suspected ADRs	47	17	64
Incident community‐acquired suspected ADRs during hospital stay	18	9	27
Serious incident community‐acquired suspected ADRs	7	2	9
Hospital‐acquired antibiotic‐assoc. suspected ADRs	33	61	94
Serious incident hospital‐acquired suspected ADRs	16	16	32
	Proportion with suspected ADRs, % (*n*/*N*)		
Characteristic at patient‐level	HIV+	HIV‐ or Unknown	Total
Overall, suspected ADRs^b^	59% (136/232)	35% (184/530)	42% (320/762)
Antibiotic‐associated community‐ or hospital‐acquired suspected ADRs			
Percent of overall sample^b^	32% (75/232)	14% (73/530)	19% (148/762)
Percent of patients who experienced at least one ADR^b^	55% (75/136)	40% (73/184)	46% (148/320)
Prevalence of antibiotic‐associated community‐acquired ADRs			
At hospital admission^b^	16% (38/232)	1% (7/530)	6% (45/762)
Among antibiotic‐users in the 4‐weeks preadmission^b^	21% (38/178)	6% (7/122)	15% (45/300)
Prevalence of serious community‐acquired ADRs^b^	13% (23/178)	1% (1/122)	8% (24/300)
Incidence of antibiotic‐associated suspected ADRs			
Incidence (overall) of community‐ or hospital‐acquired ADRs^a^, ^b^	22% (51/228)	16% (66/401)	19% (117/629)
Incidence of community‐acquired ADRs during hospital stay	10% (18/178)	7% (9/122)	9% (27/300)
Incidence of hospital‐acquired ADRs during hospital stay	15% (33/221)	16% (61/382)	16% (94/603)
Incidence (overall) of serious community/hospital‐acquired ADRs^b^	10% (23/228)	4% (16/401)	6% (39/629)
Incidence of serious community‐acquired ADRs	4% (7/178)	2% (2/122)	3% (9/300)
Incidence of serious hospital‐acquired ADRs	7% (16/221)	4% (16/382)	5% (32/603)

a Some patients experienced more than one community‐acquired or hospital‐acquired antibiotic‐associated suspected ADR with incidence overlap in four patients.

b Statistically significant difference at *P* < 0.05.

### Prevalence of community‐acquired aa‐ADRs

The prevalence of community‐acquired aa‐ADRs was 6% (45/762; 95% CI: 4–8%) of all patients or 15% (45/300; 95% CI: 11–20%) of those who had received antibiotics in the 4‐weeks preadmission. Serious prevalent aa‐ADRs were encountered by 8% (24/300; 95% CI: 5–12%) of patients who used antibiotics preadmission or by half (53%, 24/45; 95% CI: 39–68%) of those with prevalent aa‐ADRs, see Table 2.

The prevalence of aa‐ADRs among preadmission antibiotic‐users was three‐fold higher among HIV‐positive vs. HIV‐negative/unknown serostatus inpatients [21% (38/178) vs. 6% (7/122); *χ* ^2^ = 13.8; *P* < 0.001]; and 13‐fold higher for serious prevalent aa‐ADRs [13% (23/178) vs. 1% (1/122); *χ* ^2^ = 14.4; *P* < 0.001], see Table 2.

### Incidence of aa‐ADRs during hospitalization

The incidence of aa‐ADRs was 19% (117/629; 95% CI: 16–22%) of inpatients who received antibiotics [community‐acquired: 9% (27/300; 95% CI: 6–13%); hospital‐acquired: 16% (94/603; 95% CI: 13–19%)], four patients having developed incident community‐acquired and hospital‐acquired aa‐ADRs during the current hospitalization, see Tables 1, 2. Serious incident aa‐ADRs were encountered by 6% (39/629; 95% CI: 4–8%) of patients or by one‐third (33%, 39/117; 95% CI: 25–42%) of those with incident aa‐ADRs, see Table 2.

The incidence of serious community‐acquired/hospital‐acquired aa‐ADRs was two‐fold higher among HIV‐positive vs. HIV‐negative/unknown serostatus inpatients [10% (23/228) vs. 4% (16/401); *χ* ^2^ = 9.3; *P* = 0.002].

### Suspected aa‐ADRs by single antibiotic class, individual antibiotic, and system organ class

At the suspected ADR level of analysis, antibiotics contributed 41% (269/662) of all suspected ADRs (148 patients shared 269 aa‐ADRs), 44% (118/269) of which were linked to a single antibiotic class only. Ceftriaxone accounted for 43% (50/117) of aa‐ADRs attributable to individual antibiotics only, followed by metronidazole (21%, 24/117) and cotrimoxazole (13%, 15/117). Forty‐three percent (115/269) of aa‐ADRs were community‐acquired. Hospital‐acquired aa‐ADRs were more likely than community‐acquired aa‐ADRs to involve a single antibiotic class only (56% vs. 27%), see Table 3.

**Table 3:** Distribution of 269 antibiotic‐associated suspected ADRs at ADR‐level, Uganda, 2014

Characteristic at suspected ADR‐level^a^	HIV status, % (*n*/*N*)		
	HIV+	HIV‐ or Unknown	Total
Proportion of suspected ADRs that are antibiotic‐associated	43% (131/302)	38% (138/360)	41% (269/662)
Number of antibiotic classes			
One antibiotic class only^c^	34% (44/131)	54% (74/138)	44% (118/269)
Two or more antibiotic classes only^c^	10% (13/131)	19% (26/138)	15% (39/269)
One or more antibiotic class(es) + one or more other drug class(es)^c^	56% (74/131)	28% (38/138)	42% (112/269)
Individual antibiotics only b			
Ceftriaxone (J01DD04)	35% (17/49)	49% (33/68)	43% (50/117)
Metronidazole (J01XD01) & (P01AB01)	12% (6/49)	26% (18/68)	21% (24/117)
Cotrimoxazole (J01EE01)^c^	31% (15/49)	0% (0/68)	13% (15/117)
Isoniazid^c^	12% (6/49)	0% (0/68)	5% (6/117)
Ciprofloxacin (J01MA02)	2% (1/49)	7% (5/68)	5% (6/117)
Erythromycin (J01FA01)	2% (1/49)	6% (4/68)	4% (5/117)
Azithromycin (J01FA10)	0% (0/49)	7% (5/68)	4% (5/117)
Levofloxacin (J01MA12)	0% (0/49)	3% (2/68)	2% (2/117)
Ampicillin‐cloxacillin (J01CR50), Amoxicillin‐clavulanate (J01CR02)	2% (1/49)	1% (1/68)	2% (2/117)
Rifampicin	4% (2/49)	0% (0/68)	2% (2/117)
Community‐acquired or hospital‐acquired aa‐ADRs			
Proportion of community‐acquired aa‐ADRs^c^	65% (85/131)	22% (30/138)	43% (115/269)
One antibiotic class only	27% (23/85)	27% (8/30)	27% (31/115)
Two or more antibiotic classes only^c^	11% (9/85)	50% (15/30)	21% (24/115)
One or more antibiotic class(es) + one or more other drug class(es)^c^	62% (53/85)	23% (7/30)	52% (60/115)
Proportion of hospital‐acquired aa‐ADRs^c^	35% (46/131)	78% (108/138)	57% (154/269)
One antibiotic class only	46% (21/46)	61% (66/108)	56% (87/154)
Two or more antibiotic classes only	9% (4/46)	10% (11/108)	10% (15/154)
One or more antibiotic class(es) + one or more other drug class(es)^c^	46% (21/46)	29% (31/108)	36% (52/154)

a Some patients experienced more than one community‐acquired or hospital‐acquired antibiotic‐associated suspected ADRs.

b One antibiotic‐associated suspected ADR attributed to two antibiotics from the same antibiotic class (quinolones: ciprofloxacin and levofloxacin) was excluded.

c Statistically significant difference at *P* < 0.05.

Individual antibiotics with the highest frequencies of community‐acquired/hospital‐acquired aa‐ADRs were ceftriaxone (110), cotrimozaxole (58), metronidazole (54), isoniazid (35), rifampicin (26), pyrazinamide (23), ethambutol (23), levofloxacin (15), and ciprofloxacin (13), among others. Most cotrimoxazole aa‐ADRs were community‐acquired (54/58), see Table 4.

**Table 4:** Individual antibiotics most frequently implicated in causing the 269 antibiotic‐associated suspected ADRs (aa‐ADRs) among 148 of 762 hospitalized patients, Uganda, 2014

Antibiotic class, antibiotic	No. of aa‐ADRs^a^	No. of community‐acquired aa‐ADRs	No. of hospital‐acquired aa‐ADRs	DDDs used during current hospitalization	No. of Patients who used drug in hospital	No. of hospital‐acquired aa‐ADRs/100 DDDs	95% CIs^b^ of hospital‐acquired ADRs/100 DDDs
Cephalosporins							
Ceftriaxone	110	16	94	398.0	398	24	19–28
Penicillins							
Ampicillin^c^	2	0	2	8.8	11	23	0–50
Amoxicillin‐clavulanate	1	0	1	19.0	8	5	0–15
Amoxicillin	6	4	2	169.0	57	1	0–6
Ampicillin‐cloxacillin	1	1	0	24.4	32	0	0–4
Quinolones							
Levofloxacin	15	0	15	62.0	17	24	14–35
Ciprofloxacin	13	3	10	279.9	114	4	1–6
Nitroimidazole derivatives							
Metronidazole	54	10	44	309.1	246	14	10–18
Sulfonamides and trimethoprim							
Cotrimoxazole	58	54	4	358.0	162	1	0–6
Macrolide antibiotics							
Erythromycin	12	6	6	79.5	19	8	2–13
Azithromycin	8	2	6	123.3	26	5	1–9
Aminoglycosides							
Gentamicin	1	0	1	15.0	12	7	0–19
Antileprotics							
Dapsone	3	3	0	0.0	5	0	0–4
Antituberculous drugs							
Pyrazinamide	23	21	2	117.3	38	2	0–7
Isoniazid	35	33	2	155.8	49	1	0–6
Rifampicin	26	25	1	113.8	40	1	0–6
Ethambutol	23	22	1	122.8	46	1	0–6

a One or more antibiotics or other drug class(es) may have been implicated in the causation of an aa‐ADR.

b CIs is confidence intervals.

c 95% CIs are wide and include null value.

The highest incidence‐rates of hospital‐acquired aa‐ADRs, standardized by DDDs, were from ceftriaxone (24 aa‐ADRs/100 DDDs), levofloxacin (24 aa‐ADRs/100 DDDs), and metronidazole (14 aa‐ADRs/100 DDDs); though levofloxacin consumption was quite low (*n* = 17; DDDs = 62), see Table 4. Ceftriaxone showed stronger signals for hospital‐acquired aa‐ADRs than metronidazole in both HIV‐positive [(19 per 100 DDDs) vs. (10 per 100 DDDs); *P* = 0.046] and HIV‐negative/unknown status [(26 per 100 DDDs) vs. (17 per 100 DDDs); *P* = 0.014] patients, respectively, see Table S1.

The incidence of serious hospital‐acquired aa‐ADRs, standardized by DDDs, was highest for ceftriaxone (7 aa‐ADRs/100 DDDs) followed by metronidazole (3 aa‐ADRs/100 DDDs) and levofloxacin (2 aa‐ADRs/100 DDDs).

The system organ classes most frequently affected were the gastrointestinal (50%, 135/269), neurological (24%, 64/269), body‐general (10%, 27/269), and skin/appendages (6%, 17/269), among others. Serious aa‐ADRs from patients of known HIV‐positive status were significantly more frequent in the gastrointestinal [HIV‐positive: 35% (20/57) vs. HIV‐negative/unknown: 6% (5/78); *χ* ^2^ = 17.9; *P* < 0.001] and neurological [HIV‐positive: 52% (15/29) vs. HIV‐negative/unknown: 20% (7/35); *χ* ^2^ = 7.1; *P* = 0.008] systems, see Table 5.

**Table 5:** System Organ Class distribution of the 269 antibiotic‐associated suspected ADRs experienced by 148 hospitalized patients, Uganda, 2014

System Organ Class (SOC) name	No. of aa‐ADRs (% column)	No. of serious aa‐ADRs (% row)	Serious aa‐ADRs by HIV‐status, % (*n*/*N*)	
			HIV+	HIV‐ or unknown
Gastrointestinal disorders^a^	135 [50]	25 (19)	35% (20/57)	6% (5/78)
Neurological disorders^a^	64 [24]	22 (34)	52% (15/29)	20% (7/35)
Body – General disorders	27 [10]	13 (48)	60% (9/15)	33% (4/12)
Skin and appendages disorders	17 [6]	5 (29)	33% (4/12)	20% (1/5)
Others	26 [10]	21 (81)	83% (15/18)	75% (6/8)

a Statistically significant difference in proportions of serious ADRs by HIV‐status at *P* < 0.05 (gastrointestinal: *χ* ^2^ = 17.9; *P* < 0.001 & neurological: *χ* ^2^ = 7.1; *P* = 0.008).

### Causality, preventability and severity of aa‐ADRs

Only 25% (66/269) of aa‐ADRs were of probable/definite causality. Vomiting was the commonest aa‐ADR in both the possible (17%, 35/203) and probable/definite (20%, 13/66) causality categories. Loss of appetite (12%, 25/203) was the only aa‐ADR in the top 10 most frequent possible aa‐ADRs that did not feature among the top 10 most frequent probable/definite aa‐ADRs, see Table S2.

Most aa‐ADRs were preventable (64%, 171/269). However, 65% (43/66) of probable/definite aa‐ADRs were nonpreventable. Table**** 6 shows that one in seven (14%, 38/269) aa‐ADRs was severe (grade three intensity).

**Table 6:** Severity, seriousness, and rarity of 269 antibiotic‐associated suspected ADRs in hospitalized patients, Uganda, 2014

Assessment	Category	Frequency of suspected aa‐ADRs (*n*, %)^a^
Severity^a^	Mild	110 (41)
	Moderate	120 (45)
	Severe	38 (14)
	Life‐threatening	1 (0)
Serious	Yes^a^	86 (32)
	Required intervention to prevent damage^b^	48 (56)
	Caused or prolonged hospitalization^b^	25 (29)
	Other medically significant condition^b^	10 (12)
	Caused death^b^	1 (1)
	Life‐threatening^b^	1 (1)
	Caused disability^b^	1 (1)
	No^a^	183 (68)
Rare^a^	Yes	24 (9)
	No	242 (90)
	Incidence unknown	3 (1)

a Denominator used was the total number of antibiotic‐associated suspected ADRs, *n* = 269.

b Denominator used was the number of serious antibiotic‐associated suspected ADRs, *n* = 86.

### Seriousness of aa‐ADRs

Thirty‐two percent (86/269) of aa‐ADRs were judged as serious: the majority (56%, 48/86) required intervention to prevent permanent damage, and 29% (25/86) caused or prolonged hospitalization. Fatal jaundice [isoniazid], disabling itchy skin rash with numbness of lower swollen legs [ethambutol, isoniazid] and life‐threatening difficulty in breathing with shortness of breath [rifampicin] occurred in known HIV‐positive patients; see Table 7.

**Table 7:** List of 86 serious antibiotic‐associated suspected adverse drug reactions experienced by hospitalized patients, Uganda, 2014

Adverse Drug Reaction	Drug	Severity	Causality	Rarity	HIV‐status	Community or Hospital‐acquired	System Organ Class	Grade of Seriousness
FEVER	INH, RIFAMPICIN, PYRAZINAMIDE	Moderate	Possible	No	Negative	Community‐acquired	Body ‐ General	Required intervention to prevent damage
ANEMIA	UNKNOWN HERBAL, CEFTRIAXONE, CAPTOPRIL	Moderate	Possible	No	Negative	Community‐acquired	Blood	Required intervention to prevent damage
FEVER	CEFTRIAXONE	Severe	Possible	No	Negative	Hospital‐acquired	Body ‐ General	Required intervention to prevent damage
DIZZINESS	CEFTRIAXONE	Severe	Possible	No	Negative	Hospital‐acquired	Neurological	Required intervention to prevent damage
VOMITING	CEFTRIAXONE, TRAMADOL	Severe	Possible	No	Negative	Hospital‐acquired	Gastrointestinal	Required intervention to prevent damage
FEVER	CEFTRIAXONE	Severe	Possible	No	Negative	Hospital‐acquired	Body ‐ General	Required intervention to prevent damage
DIZZINESS	CEFTRIAXONE, METRONIDAZOLE	Moderate	Possible	No	Negative	Hospital‐acquired	Neurological	Required intervention to prevent damage
PERIPHERAL NEUROPATHY	LEVOFLOXACIN	Moderate	Possible	No	Negative	Hospital‐acquired	Neurological	Caused or prolonged Hosp
TACHYCARDIA	CIPROFLOXACIN	Moderate	Possible	No	Negative	Hospital‐acquired	Cardiovascular	Required intervention to prevent damage
DIZZINESS	METRONIDAZOLE, AMOXICILLIN	Severe	Possible	NK	Negative	Hospital‐acquired	Neurological	Caused or prolonged Hosp
WORSENED JAUNDICE	CIPROFLOXACIN, CEFTRIAXONE, METRONIDAZOLE	Moderate	Possible	No	Negative	Hospital‐acquired	Liver and biliary	Other medically significant condition
CONVULSIONS ‐ GTC (2 EPISODES)	METRONIDAZOLE	Severe	Possible	No	Negative	Hospital‐acquired	Neurological	Required intervention to prevent damage
VOMITING	CEFTRIAXONE	Severe	Possible	No	Negative	Hospital‐acquired	Gastrointestinal	Other medically significant condition
ORAL SORES	CEFTRIAXONE	Moderate	Possible	No	Negative	Hospital‐acquired	Gastrointestinal	Other medically significant condition
HYPERTENSION	CIPROFLOXACIN	Severe	Probable	No	Negative	Hospital‐acquired	Cardiovascular	Required intervention to prevent damage
PARESTHESIA	CEFTRIAXONE	Moderate	Probable	NK	Negative	Hospital‐acquired	Neurological	Other medically significant condition
ITCHING SKIN ‐ MULTIFORME RASH	DICLOFENAC, METRONIDAZOLE	Moderate	Probable	No	Negative	Hospital‐acquired	Skin and appendages	Required intervention to prevent damage
PARESTHESIAS (PERIPHERAL NEUROPATHY)	HRZE	Severe	Possible	No	Unknown	Community‐acquired	Neurological	Required intervention to prevent damage
HIGH GRADE FEVER WITH CHILLS AND RIGOR	HRZE	Severe	Possible	No	Unknown	Community‐acquired	Body ‐ General	Caused or prolonged Hosp
BLURRED VISION	METRONIDAZOLE	Mild	Possible	No	Unknown	Hospital‐acquired	Vision	Other medically significant condition
VOMITING (3 EPISODES)	CEFTRIAXONE, TRAMADOL	Mild	Possible	No	Unknown	Hospital‐acquired	Gastrointestinal	Other medically significant condition
LOSS OF APPETITE	METRONIDAZOLE	Moderate	Possible	No	Unknown	Hospital‐acquired	Gastrointestinal	Caused or prolonged Hosp
DECREASED URINE OUTPUT	CEFTRIAXONE	Severe	Probable	Yes	Unknown	Hospital‐acquired	Urinary tract	Other medically significant condition
LOSS OF APPETITE	CTX, METRONIDAZOLE	Moderate	Possible	No	Positive	Community‐acquired	Gastrointestinal	Required intervention to prevent damage
PARESTHESIAS	3TC, CTX	Moderate	Possible	No	Positive	Community‐acquired	Neurological	Other medically significant condition
FEVER	AZT/3TC/EFV, CTX	Severe	Possible	No	Positive	Community‐acquired	Body ‐ General	Required intervention to prevent damage
SEVERE PALLOR OF MUCUS MEMBRANES	HRZE, TDF/3TC/NVP, CTX	Severe	Possible	No	Positive	Community‐acquired	Blood	Required intervention to prevent damage
DIZZINESS	HRZE, TDF/3TC, CTX	Severe	Possible	No	Positive	Community‐acquired	Neurological	Caused or prolonged Hosp
PRODUCTIVE COUGH	CTX, TDF/3TC	Severe	Possible	No	Positive	Community‐acquired	Respiratory	Required intervention to prevent damage
JAUNDICE	HRZE, TDF/3TC, CTX	Moderate	Possible	No	Positive	Community‐acquired	Liver and biliary	Required intervention to prevent damage
DIFFICULTY IN BREATHING	RIFAMPICIN, TDF/3TC, CTX	Severe	Possible	No	Positive	Community‐acquired	Respiratory	Required intervention to prevent damage
NUMBNESS OF BOTH LOWER LIMBS	HE, 3TC, CTX	Moderate	Possible	No	Positive	Community‐acquired	Neurological	Required intervention to prevent damage
DIZZINESS	COARTEM, DAPSONE, FLUCONAZOLE	Moderate	Possible	No	Positive	Community‐acquired	Neurological	Required intervention to prevent damage
PARAPARESIS	INH	Moderate	Possible	No	Positive	Community‐acquired	Neurological	Caused or prolonged Hosp
JAUNDICE	INH	Life‐threatening	Possible	No	Positive	Community‐acquired	Liver and biliary	Caused death
GENERALIZED MACULO‐PAPULAR RASH	CTX, ARVS (TDF/3TC/EFV)	Moderate	Possible	No	Positive	Community‐acquired	Skin and appendages	Caused or prolonged Hosp
VOMITING	FLUCONAZOLE, ACICLOVIR, CEFTRIAXONE	Moderate	Possible	Yes	Positive	Community‐acquired	Gastrointestinal	Caused or prolonged Hosp
SEVERE ANEMIA 3.4G/DL	TDF/3TC, CTX	Severe	Possible	No	Positive	Community‐acquired	Blood	Caused or prolonged Hosp
WORSENED PALLOR	TDF/3TC, CTX	Severe	Possible	No	Positive	Community‐acquired	Skin and appendages	Required intervention to prevent damage
DIARRHEA	TDF/3TC/EFV, CTX	Moderate	Possible	No	Positive	Community‐acquired	Gastrointestinal	Caused or prolonged Hosp
HEADACHE	CTX	Moderate	Possible	No	Positive	Community‐acquired	Neurological	Caused or prolonged Hosp
VOMITING	HRZE	Severe	Possible	No	Positive	Community‐acquired	Gastrointestinal	Caused or prolonged Hosp
ANEMIA	CTX	Moderate	Possible	No	Positive	Community‐acquired	Blood	Caused or prolonged Hosp
ABDOMINAL DISCOMFORT/DISTENTION	HRZE, AZT	Moderate	Possible	No	Positive	Community‐acquired	Gastrointestinal	Other medically significant condition
PARESTHESIAS	CTX, TDF/3TC/LPV/RTV	Moderate	Possible	No	Positive	Community‐acquired	Neurological	Required intervention to prevent damage
PERSISTENT DIARRHOEA	3TC/TDF/EFV, CTX	Severe	Possible	No	Positive	Community‐acquired	Gastrointestinal	Caused or prolonged Hosp
VOMITING	3TC/TDF/EFV, CTX	Severe	Possible	No	Positive	Community‐acquired	Gastrointestinal	Required intervention to prevent damage
ANOREXIA	3TC/TDF, CTX	Moderate	Possible	No	Positive	Community‐acquired	Gastrointestinal	Required intervention to prevent damage
JOINT PAIN	3TC/TDF, CTX	Severe	Possible	No	Positive	Community‐acquired	Musculoskeletal	Required intervention to prevent damage
DEEP JAUNDICE	RHZ, AZT/3TC	Severe	Possible	No	Positive	Community‐acquired	Liver and biliary	Required intervention to prevent damage
PERIPHERAL NEUROPATHY	AZT, 3TC, INH	Moderate	Possible	No	Positive	Community‐acquired	Neurological	Other medically significant condition
COUGH	TDF/3TC, CTX	Severe	Possible	No	Positive	Community‐acquired	Respiratory	Required intervention to prevent damage
DRY COUGH WITH SHORTNESS OF BREATH	CTX	Severe	Possible	Yes	Positive	Community‐acquired	Respiratory	Required intervention to prevent damage
DIZZINESS	METRONIDAZOLE	Severe	Possible	No	Positive	Community‐acquired	Neurological	Required intervention to prevent damage
DIZZINESS	TDF/3TC, RHZE	Moderate	Probable	No	Positive	Community‐acquired	Neurological	Required intervention to prevent damage
LOSS OF APPETITE	3TC, TDF, RIFAMPICIN, PYRAZINAMIDE	Moderate	Probable	No	Positive	Community‐acquired	Gastrointestinal	Required intervention to prevent damage
VOMITING	HRZE	Moderate	Probable	No	Positive	Community‐acquired	Gastrointestinal	Required intervention to prevent damage
GENERALIZED BODY WEAKNESS	TDF/3TC, RIFAMPICIN	Moderate	Probable	No	Positive	Community‐acquired	Body ‐ General	Caused or prolonged Hosp
HEADACHE	INH, RIFAMPICIN	Moderate	Probable	No	Positive	Community‐acquired	Neurological	Required intervention to prevent damage
CONSTIPATION	INH	Moderate	Probable	No	Positive	Community‐acquired	Gastrointestinal	Required intervention to prevent damage
PRURITUS	TDF/EFV, METRONIDAZOLE, INH, ETHAMBUTOL	Severe	Probable	No	Positive	Community‐acquired	Skin and appendages	Caused or prolonged Hosp
ITCHY RASH WITH NUMBNESS OF LOWER SWOLLEN LEGS	HE, CARVEDILOL	Moderate	Probable	No	Positive	Community‐acquired	Skin and appendages	Caused disability
JAUNDICE	CTX	Moderate	Probable	No	Positive	Community‐acquired	Liver and biliary	Required intervention to prevent damage
PERIPHERAL NEUROPATHY	3TC, METRONIDAZOLE	Moderate	Probable	Yes	Positive	Community‐acquired	Neurological	Caused or prolonged Hosp
DIB WITH SHORTNES OF BREATH	RIFAMPCIN	Severe	Probable	No	Positive	Community‐acquired	Respiratory	Life‐threatening
ANEMIA	CTX, TDF/3TC/LPV/RTV	Moderate	Probable	No	Positive	Community‐acquired	Blood	Caused or prolonged Hosp
PEDAL EDEMA	RIFAMPICIN	Severe	Probable	No	Positive	Community‐acquired	Cardiovascular	Required intervention to prevent damage
PARESTHESIA	HRZE	Mild	Probable	No	Positive	Community‐acquired	Neurological	Caused or prolonged Hosp
VOMITING	CEFTRIAXONE, METRONIDAZOLE, BLOOD, DICLOFENAC	Severe	Possible	No	Positive	Hospital‐acquired	Gastrointestinal	Required intervention to prevent damage
DIZZINESS	CEFTRIAXONE, METRONIDAZOLE, DICLOFENAC	Moderate	Possible	No	Positive	Hospital‐acquired	Neurological	Required intervention to prevent damage
FEVER	CTX, TDF/3TC/EFV	Moderate	Possible	No	Positive	Hospital‐acquired	Body ‐ General	Required intervention to prevent damage
FEVER	CEFTRIAXONE	Moderate	Possible	No	Positive	Hospital‐acquired	Body ‐ General	Required intervention to prevent damage
VOMITING	CEFTRIAXONE, TDF/3TC/EFV	Severe	Possible	No	Positive	Hospital‐acquired	Gastrointestinal	Caused or prolonged Hosp
DIZZINESS	CEFTRIAXONE, METOCLOPRAMIDE	Moderate	Possible	No	Positive	Hospital‐acquired	Neurological	Caused or prolonged Hosp
VOMITING	CEFTRIAXONE	Moderate	Possible	No	Positive	Hospital‐acquired	Gastrointestinal	Caused or prolonged Hosp
FEVER 39.6C	CEFTRIAXONE	Severe	Possible	No	Positive	Hospital‐acquired	Body ‐ General	Caused or prolonged Hosp
FEVER 38.3C	ACICLOVIR, CEFTRIAXONE	Severe	Possible	No	Positive	Hospital‐acquired	Body ‐ General	Caused or prolonged Hosp
ANOREXIA	CTX	Moderate	Possible	No	Positive	Hospital‐acquired	Gastrointestinal	Required intervention to prevent damage
DIARRHEA	CEFTRIAXONE	Moderate	Possible	No	Positive	Hospital‐acquired	Gastrointestinal	Required intervention to prevent damage
SEVERE ABDOMINAL PAIN	CIPROFLOXACIN	Severe	Possible	No	Positive	Hospital‐acquired	Gastrointestinal	Required intervention to prevent damage
VOMITING	CEFTRIAXONE	Severe	Possible	No	Positive	Hospital‐acquired	Gastrointestinal	Caused or prolonged Hosp
HIGH GRADE FEVER 39.2C	CEFTRIAXONE	Severe	Possible	No	Positive	Hospital‐acquired	Body ‐ General	Required intervention to prevent damage
VOMITING	ERYTHROMYCIN, CEFTRIAXONE	Severe	Probable	No	Positive	Hospital‐acquired	Gastrointestinal	Required intervention to prevent damage
FEVER 38.5C	CEFTRIAXONE	Moderate	Probable	No	Positive	Hospital‐acquired	Body ‐ General	Required intervention to prevent damage
FEVER 38 C	CEFTRIAXONE, DUOVIR‐N, INH, PYRAZINAMIDE	Mild	Probable	No	Positive	Hospital‐acquired	Body ‐ General	Required intervention to prevent damage
VOMITING	CEFTRIAXONE	Moderate	Probable	No	Positive	Hospital‐acquired	Gastrointestinal	Required intervention to prevent damage

CTX, Cotrimoxazole; GTC, General Tonic‐Clonic; INH, Isoniazid; HE, Isoniazid/Ethambutol; Hosp, Hospital; RHZ, Rifampicin/Isoniazid/Pyrazinamide; HRZE, Isoniazid/rifampicin/pyrazinamide/ethambutol; AZT, Zidovudine; TDF, Tenofovir; 3TC, lamivudine; NVP, nevirapine; EFV, Efavirenz; LPV, Lopinavir; RTV, Ritonavir; ARVS, Antiretrovirals; DUOVIR‐N, Zidovudine/lamivudine/nevirapine; NK, Incidence unknown; DIB, Difficulty in Breathing.

Seventy‐three percent (63/86) of serious aa‐ADRs were from HIV‐positive patients, mostly community‐acquired (71%, 45/63): with cotrimoxazole (24/45) and antituberculous drugs [(22/45): isoniazid (17/45), rifampicin (15/45), ethambutol (11/45), and pyrazinamide (10/45)] being the most frequently implicated. The loss of appetite (4), cough (4), peripheral neuropathy (3), and anemia (3) linked to cotrimoxazole use occurred thrice or more and, similarly, peripheral neuropathy (4) and jaundice (3) for antituberculous drugs.

Ceftriaxone was the most frequently linked to serious hospital‐acquired aa‐ADRs [70% (26/37); HIV‐positive: 83% (15/18) & HIV‐negative/unknown status: 58% (11/19)]: the commonest being fever (6) and vomiting (6), see Tables 7, S3.

### Allergic aa‐ADRs

Allergic reactions occurred in 22% (32/148, 95% CI: 15–29%) of patients who experienced aa‐ADRs [community‐acquired: 19% (12/62; 95% CI: 10–31%); hospital‐acquired: 26% (24/94; 95% CI: 17–36%)]: 14 (44%) of 32 allergic reactions were serious.

A quarter of the patients who were admitted with at least one ADR related to any medication class had prevalent community‐acquired aa‐ADRs. (15/62; 24%, 95% CI: 14–37%).

### Rarity of aa‐ADRs

Nine percent (24/269) of aa‐ADRs, see Tables 7, S4, experienced by 22 patients were rare. Twelve rare aa‐ADRs were attributed to metronidazole, those in *italics* being of the *central nervous system* [*dizziness* (4), constipation (3), *convulsions* (1), *blurred vision* (1), facial itchy skin rash (1), fever (1), and *headache* (1)]; and six to cotrimoxazole [anorexia (3), jaundice (1), dry cough with shortness of breath (1), and anemia (1)]. Nine of the 24 rare aa‐ADRs were serious: four linked to cotrimoxazole (jaundice, anorexia, anemia, and dry cough with shortness of breath), three to metronidazole (*blurred vision, convulsions, and dizziness*), one to levofloxacin (peripheral neuropathy), and one to ceftriaxone (decreased urine output). Moderate jaundice (cotrimoxazole) and severe decreased urine output (ceftriaxone) were nonpreventable rare and serious aa‐ADRs of probable causality, whereas the rest of the rare and serious aa‐ADRs were of possible causality, see Table S4. The jaundice linked to cotrimoxazole occurred in a 33‐year‐old HIV‐positive female who had not yet commenced antiretroviral therapy; the patient's other working diagnoses were acute gastroenteritis and dysentery. The severe decreased urine output linked to ceftriaxone occurred in an 18‐year‐old HIV‐positive male not yet on antiretroviral therapy whose other working diagnosis was bronchopneumonia: This patient's final diagnosis was acute kidney injury.

Half the rare aa‐ADRs (12/24) were community‐acquired, eight of which were cotrimoxazole‐linked and four metronidazole‐linked. Eight of the 12 hospital‐acquired rare aa‐ADRs were attributed to metronidazole use and three to ceftriaxone, see Table S4.

## Discussion

Suspected aa‐ADRs were common both at admission and during hospital stay. The incidence of aa‐ADRs in our study was threefold higher than that observed in a French cohort of 3963 hospitalized patients (Courjon et al. 2013) which could, in part, be linked to the higher consumption of antibiotics by our inpatients for HIV‐associated comorbidities. Most of our inpatients received antibiotics whose use was significantly higher among HIV‐infected patients (Kiguba et al. 2016a). The medical wards showed a higher HIV‐seroprevalence (38%) than gynecology where HIV‐seroprevalence (9%) was similar to the national estimate (7.3%) (Uganda AIDS Commission, 2015). The fact that most patients received antibiotics makes our study epidemiologically efficient. Few aa‐ADRs would be observed in a hospitalized‐population in which antibiotic prescribing was infrequent. Of all suspected ADRs attributed to any medication class, a large proportion (one in three) was linked to antibiotics, which corroborates findings from South Africa, a setting with a similarly high HIV/AIDS burden, where one in five ADRs (11/51) was antibiotic‐associated (Mehta et al. 2008).

Ceftriaxone showed the strongest signal for hospital‐acquired aa‐ADRs, even among HIV‐negative/unknown serostatus patients, which could be a reflection of ceftriaxone's high prescription rate in our hospital setting. Strong signals were also shown by metronidazole and levofloxacin (based on small numbers of patients for levofloxacin). Unmasking of the signal from the infrequently used levofloxacin demonstrates the utility of standardizing absolute aa‐ADRs by DDDs administered (Hamad et al. 2013). However, much larger well‐designed epidemiological studies in Uganda and other similar resource‐limited settings could provide more clues on the aa‐ADR profiles of ceftriaxone and levofloxacin, which are otherwise generally known to be safe. We did not test the quality of the antibiotics used and hence how this impacted on the incidence of hospital‐acquired aa‐ADRs by individual antibiotics. Nonetheless, this study has identified individual antibiotics (ceftriaxone, metronidazole, levofloxacin) with potentially higher risk for hospital‐acquired aa‐ADRs. The identified antibiotics can be prioritized for routine pharmaceutical quality assessment in resource‐limited settings where the challenge of counterfeit and substandard drugs prevails (Almuzaini et al. 2013).

Community‐acquired aa‐ADRs were mostly linked to the preadmission use of cotrimoxazole for prevention of, and antituberculous drugs for treatment of, HIV‐associated opportunistic infections. The cotrimoxazole link to community‐acquired aa‐ADRs is in accordance with previous research in the US (Shehab et al. 2008) though cotrimoxazole is also a common cause of hospital‐acquired aa‐ADRs in France (Courjon et al. 2013). Similarly, antituberculous drugs are a major cause of hospital‐acquired aa‐ADRs in South Africa (Mehta et al. 2008), though most in our study were community‐acquired.

We document a many‐fold higher risk for serious prevalent aa‐ADRs at the time of hospital admission in known HIV‐positive patients. Our study also corroborates findings from the United Kingdom, South Africa, and Rwanda which reported that the incidence of serious community‐acquired‐/hospital‐acquired aa‐ADRs in HIV‐infected patients is at least twice that in HIV‐uninfected patients (Breen et al. 2006; Marks et al. 2009; Lorent et al. 2011), probably due to drug‐drug interactions from concomitant antiretroviral medications (Breen et al. 2006). Moreover, Marks et al. (2009) have argued that the higher incidence of serious aa‐ADRs in South African patients on antituberculous treatment could have been predominated by their HIV‐infection rather than by coadministered antiretroviral therapy.

One in four ADRs related to hospital admission was linked to antibiotic use, which is similar to what is reported in the US (Shehab et al. 2008), but higher than as observed in Spain (Carrasco‐Garrido et al. 2010), The Netherlands (van der Hooft et al. 2008), and India (Sonal et al. 2011). The fatal, disabling or life‐threatening serious community‐acquired aa‐ADRs linked to individual antituberculous drugs highlight the need for vibrant pharmacovigilance of antituberculous drugs to safeguard patients in our setting where treatment discontinuation or modification may be warranted. The proportion of allergic aa‐ADRs in our cohort (22%), two‐fifths of them serious, was much lower than is reported in the US (79%) (Shehab et al. 2008), probably by reason of differences in study methodology. Our estimate is based on representatively sampled patients as the unit of analysis, whereas the US study used hospital visits for a drug‐related ADR.

The majority of rare and serious aa‐ADRs were accounted to cotrimoxazole and metronidazole use, yet the number of inpatients who received ceftriaxone during hospital stay was twofold higher than for metronidazole and cotrimoxazole. The aa‐ADRs linked to cotrimoxazole use were probably as a result of long‐term exposure to cotrimoxazole prophylaxis in HIV/AIDS patients. Thus, ongoing close monitoring and documentation of cotrimoxazole's safety profile in our setting is critical. Metronidazole and ceftriaxone were mostly initiated during the current admission. The rare aa‐ADRs linked to metronidazole use frequently involved the central nervous system: metronidazole is a small lipophilic molecule that can easily cross the blood–brain barrier and act centrally (Nau et al. 2010). The high incidence of metronidazole‐associated rare aa‐ADRs during hospital stay should be investigated further. Three percent (8/246; 95% CI: 1.4% to 6.4%) of patients who received metronidazole while in hospital developed a rare hospital‐acquired metronidazole‐associated aa‐ADR. Metronidazole has a BNF list of 22 rare aa‐ADRs (British National Formulary, 2014). If such aa‐ADRs occur independently within‐patient, the potential rate of occurrence of rare aa‐ADRs among metronidazole users is 22*1/1000, or 2%. The observed occurence rate of 3% for any rare aa‐ADR is thus consistent with metronidazole being associated with a long list of 22 rare aa‐ADRs.

We suggest that, periodically, Mulago Hospital and possibly other secondary care health facilities in resource‐poor settings could set up ward‐tailored ADR tracking logs, as used in our study, which are formally placed, by default, into patients' hospital files to log and retrieve, for analysis, ADRs experienced by inpatients. The ADR tracking log has all the data fields of the full suspected ADR reporting form (Form S1) and is efficient for data collection with reduced paper burden. On patient‐discharge, a copy of the log ought to be made for central analysis after every 200–500 such forms have been collected. For health facilities which admit 70–105 patients per week, a 6‐week surveillance period once every 6 months, would suffice. This approach, if well‐coordinated through a local hospital‐based pharmacovigilance unit, will give facility‐specific estimates of antibiotic safety and validate the findings of our study at a much lower cost.

This study had limitations. First, the demanding nature of data collection and high staff costs for high‐quality data limited the number of inpatients studied to the range 600–800 rather than 1200–1500 as initially intended. However, higher than anticipated prevalence at admission and incidence during hospital stay of aa‐ADRs meant that the achieved sample size was sufficient to describe the characteristics of these aa‐ADRs. Second, variations from the planned systematic random sampling approach for recruiting inpatients were minor and unlikely to alter our findings. Third, refusal rates by omitted inpatients were not formally recorded but were low. Fourth, consensus – rather than individual assessments of aa‐ADR causality and preventability followed by appraisal of interobserver agreement – was used. Fifth was the challenge of assessing ADR causality in acutely unwell patients with multiple comorbidities and on multiple medications. Hence, we screened patients using an ADR trigger tool to increase the probability to detect aa‐ADRs; and subsequently applied the Naranjo ADR scale to the suspect clinical signs and symptoms to identify those that were at least *possibly* antibiotic‐associated. We held extensive ward‐team discussions and made consultations with the literature before reaching consensus on ADR causality. Sixth, we cannot claim generalizability of our findings to district hospitals, clinics, and other lower level health centers in Uganda. Similar studies conducted in lower level health facilities could elucidate the extent and characteristics of aa‐ADRs in those settings.

## Conclusion

Ceftriaxone showed the highest risk for hospital‐acquired aa‐ADRs (also of serious hospital‐acquired aa‐ADRs), even in HIV‐negative/unknown serostatus patients, probably due to its high consumption. Levofloxacin signaled as high‐risk for hospital‐acquired aa‐ADRs despite its low frequency of in‐hospital use, thus, highlighting the utility of standardizing absolute aa‐ADR counts by DDDs administered. Cotrimoxazole and antituberculous drugs were the most frequently implicated in community‐acquired aa‐ADRs (also in serious community‐acquired aa‐ADRs). Pharmaceutical quality testing of implicated antibiotics in Uganda could be worthwhile. In sentinel settings, periodic on‐ward collection and analysis of antibiotic‐safety‐data standardized by DDDs, such as during a 6‐week surveillance period once every 6 months, should be an efficient method of tracking antibiotics with 1%‐risk for serious aa‐ADRs.

## Author Contribution

RK conceived of the study and drafted the manuscript and, in conjunction with SMB, participated in its design, implementation, statistical analysis, and drawing of inferences. CK participated in study design and, together with SMB, took part in the manuscript writing process. All authors approved the final manuscript. [3,901 words].

## Disclosure

All authors have completed the Unified Competing Interest form at http://www.icmje.org/coi_disclosure.pdf (available on request from the corresponding author). RK gratefully acknowledges funding support from the Training Health Researchers into Vocational Excellence (THRiVE) in East Africa grant number 087540, funded by the Wellcome Trust; and an African Doctoral Dissertation Research Fellowship (ADDRF) award 2013 ‐ 2015 ADF 006 by the African Population and Health Research Centre (APHRC) in partnership with the International Development Research Centre (IDRC). In UK, SMB is funded by Medical Research Council program number MC_U105260794. SMB has GSK shares. The work here reported is solely the responsibility of the authors and does not necessarily represent the official views of the supporting offices. The funders had no role in the decisions on what and where to publish.

## Supporting information


**Form S1.** Suspected ADR reporting form.

**Table S1.** (A) Incidence of hospital‐acquired antibiotic‐associated suspected ADRs per 100 Defined Daily Doses stratified by HIV‐status.(B) Incidence of hospital‐acquired antibiotic‐associated suspected ADRs per 100 patients at risk stratified by HIV‐status.

**Table S2.** Frequencies of individual antibiotic‐associated ADRs in the Probable or Definite vs. Possible ADR causality categories, Uganda, 2014.

**Table S3.** Serious antibiotic‐associated suspected adverse drug reactions, with preventability, experienced by hospitalized patients, Uganda, 2014.

**Table S4.** List of 24 rare antibiotic‐associated suspected adverse drug reactions experienced by hospitalized patients, Uganda, 2014.
