# Supplementary material for: Antibiotic‐associated suspected adverse drug reactions among hospitalized patients in Uganda: a prospective cohort study
Source: Pharmacol Res Perspect. 2017 Feb 17;5(2):e00298. doi: 10.1002/prp2.298 (PMC5368962; doi:10.1002/prp2.298)
Supplement: Supplementary file 1 — Form S1. Suspected ADR reporting form. [file PRP2-5-e00298-s001.doc]

| **Form S1: Suspected ADR Reporting Form** | |
| --- | --- |
| **Ward:** pre-fill  **Patient Number:** pre-fill **Investigator:** pre-fill | |
| **SOCIO-DEMOGRAPHIC CHARACTERISTICS** | |
| 1. **Patient surname Other names**   ……………………… …………………….. | 1. **Gender**  [1] Male [2] Female |
| 1. **Patient’s age (in completed years)?** ……….. | 1. **Patient’s telephone contact:** …………………………… |
| 1. **Body weight in Kgs (if known)**……… | 1. **Height in cms (if known)**................... |
| 1. **Village of patient’s residence:**   ……………………………………………………….. | 1. **Does patient have any history of allergies to drugs?**   [1] No [2] Yes [3] Don’t know |
| **CLINICAL CHARACTERISTICS** | |
| 1. **Date of patient’s admission to THIS WARD**   day [ ] [ ] month [ ][ ] year [ 2 ][ 0 ][ 1 ][ ] | 1. **Cause of admission**   …………………………………………………………………………………………………………………………………… |
| 1. **What is the working diagnosis?** (*List all others too*)   Working diagnosis ................................................................................................  Others....................................................................................................................................................................................... | 1. **What is the patient’s HIV serostatus?**   [1] Negative [2] Positive [3] Don’t know |
| 1. **Describe any major co-morbidities which complicate patient-care (E.g. Liver disease, Kidney disease, Allergies, Pregnancy)**………………………………………………………………………………………………………………………………………………………………………………………………………………………………………………………………………………………………………………………………………………………………………………………………………………………… | |
| **SUSPECTED ADR CHARACTERISTICS** | |
| 1. **Describe the patient’s suspected Adverse Drug Reaction (ADR):** (*Provide details of drug class, route of administration, severity, and body site/system involved, among others)*   ……………………………………………………………………………………………………………………………………………………………………………………………………………………………………………………………………………………………………………………………………………………………………………………………………………………………………… | |
| 1. **Do you consider the reaction(s) to be serious?**   [1] No  [2] Yes, please specify by ticking *all* reasons that apply:  [a] Patient died due to reaction (*record date of death*): day [ ] [ ] month [ ][ ] year [ 2 ][ 0 ][ 1 ][ ]  [b] Was life-threatening  [c] Caused or prolonged hospitalization  [d] Caused disability  [e] Required intervention to prevent permanent damage  [f] Medically significant; Give details.......................... | |
| 1. **Please describe the patient’s suspected ADR. Tick *all* descriptors that apply:**   [a] Anaphylaxis [f] Diarrhoea  [b] Skin reaction [g] Convulsions  [c] Headache [h] Nausea/vomiting  [d] Shock/collapse [I ] Injection site abscess  [e] Severe local reaction [j ] Other, Specify …………. | 1. **Type of suspected ADR:** (*Tick one only*)   [1] Allergic Reaction [2] Cardiovascular [3] Dermatologic [4] CNS/Nervous System [5] Haematological [6] Respiratory [7] Hepatic [8] Renal [9] Blood Loss [10] Hypoglycaemia [11] Gastrointestinal [12] Other, Specify……………… |
| 1. **Date on which ADR started (according to patient):**   day [ ] [ ] month [ ][ ] year [ 2 ][ 0 ][ 1 ][ ] | 1. **Patient’s location at ADR-start:**   [1] THIS WARD [2] Elsewhere in Hospital [3] Pre-admission |
| 1. **Date patient’s ADR was identified by Health worker(s):**   day [ ] [ ] month [ ][ ] year [ 2 ][ 0 ][ 1 ][ ] | 1. **Outcome of patient’s ADR:** (*Tick one only*)   [1] ADR resolved: day [ ] [ ] month [ ][ ] year [ 2 ][ 0 ][ 1 ][ ]  [2] Ongoing  [3] Death: day [ ] [ ] month [ ][ ] year [ 2 ][ 0 ][ 1 ][ ]  [4] Unknown |
| 1. **If the ADR existed pre-admission, did the ADR cause admission?**   [1] No [2] Yes [3] Don’t know | 1. **Management of the suspected ADR (***Tick all that apply***)**   [a] No Action Taken [d] Drug(s) Discontinued [b] Drug(s) Dose Reduced [e] Supportive Treatment  [c] Antidote/Antagonist Used [f] Other…………………………. |
| 1. Additional relevant information, if known (E.g. Laboratory test results & dates, discharge summaries e.t.c.)   ……………………………………………………………………………………………………………………………………………………………………………………………………………………………………………………………………………………………… | |

| **No.** | **Drug Name (Brand/Generic)** | | **Involved in ADR?**  **1=No**  **2=primary suspect, 3=secondary suspect** | **Batch/Lot No.** | | **How obtained?**  **1=Prescribed* 2=OTC^+^**  **3=Herbal** | **Expiry Date** | | **Dosage Form** | | **Dose: [Is dose within recommended range?**  **1=No**  **2=Yes** | **Frequency (E.g. 8hrly)** | | | **Route used** | **Diluent used**  **1=No, 2=Yes**  **[If yes, specify]** | | **Therapy Dates (If unknown give duration)** | | | **Reason for Use or**  **Prescribed for (Includes “To Resolve ADR”)** | | **Pre-existing contraindication in this patient?**  **1=No**  **2 = Yes**  **[If yes, specify]** |
| --- | --- | --- | --- | --- | --- | --- | --- | --- | --- | --- | --- | --- | --- | --- | --- | --- | --- | --- | --- | --- | --- | --- | --- |
|  |  |  |  |  |  |  |  |  |  |  |  |  |  |  |  |  |  | **Date drug started**  **(dd/mm)** | **Date drug stopped (dd/mm)** | |  |  |  |
| 1 |  | |  |  | |  |  | |  | | Dose: |  | | |  | Specify:  ………………. | |  |  | |  | | | Specify:  ……………………. | | --- | |
|  |  |  |  |  |  |  |  |  |  |  | Range: |  |  |  |  |  |  |  |  |  |  |  |  |
| 2 |  | |  |  | |  |  | |  | | Dose: |  | | |  | Specify:  ……………….. | |  |  | |  | | | Specify:  ……………………. | | --- | |
|  |  |  |  |  |  |  |  |  |  |  | Range: |  |  |  |  |  |  |  |  |  |  |  |  |
| 3 |  | |  |  | |  |  | |  | | Dose: |  | | |  | Specify  ……………….. | |  |  | |  | | | Specify:  ……………………. | | --- | |
|  |  |  |  |  |  |  |  |  |  |  | Range: |  |  |  |  |  |  |  |  |  |  |  |  |
| 4 |  | |  |  | |  |  | |  | | Dose: |  | | |  | Specify:  ……………….. | |  |  | |  | | | Specify:  …………………… | | --- | |
|  |  |  |  |  |  |  |  |  |  |  | Range: |  |  |  |  |  |  |  |  |  |  |  |  |
| 5 |  | |  |  | |  |  | |  | | Dose: |  | | |  | Specify:  ……………….. | |  |  | |  | | Specify:  ……………………. |
|  |  |  |  |  |  |  |  |  |  |  | Range: |  |  |  |  |  |  |  |  |  |  |  |  |
| **If known, please complete the following:** | | | | | | | | | | | | | | | | | | | | | | |  |
| **No. as per Q28 above** | | 1. **Reaction subsided after drug was stopped or dose reduced** | | | | | | | | | | | 1. **Reaction reappeared after drug was reintroduced** | | | | | | | | | |  |
|  |  | Yes | No | | Unknown | | | NA^±^ | | Reduced Dose | | |  | Yes | | | No | | | Unknown | | NA^±^ | If reintroduced, dose |
| 1 | |  |  | |  | | |  | |  | | |  |  | | |  | | |  | |  |  |
| 2 | |  |  | |  | | |  | |  | | |  |  | | |  | | |  | |  |  |
| 3 | |  |  | |  | | |  | |  | | |  |  | | |  | | |  | |  |  |
| 4 | |  |  | |  | | |  | |  | | |  |  | | |  | | |  | |  |  |
| 5 | |  |  | |  | | |  | |  | | |  |  | | |  | | |  | |  |  |

1. **Please complete the following table on ADR-suspected drugs in use by, or administered to this patient.**

*For all prescribed drugs, THIS WARD’s pharmacy will check for you if prescribed drugs were dispensed by THIS WARD’s pharmacy, or interview the patients/caregivers

^+^Over-the-counter medicine; ±Not applicable - reaction might have subsided despite not changing the dose.

**Other Drugs in use by patient (including self-medication and herbal remedies)**

| 1. **Number of ADR-implicated medicines used by, or administered to patient throughout ADMISSION: (***Fill in after completing the table on page 2***): [ ][ ]** | 1. **Total number of medicines used by, or administered to patient throughout ADMISSION: [ ][ ]** |
| --- | --- |

**ADR- Reporter’s surname:** . . . . . . . . . . . . . . . . . .. . . . . . **Designation:** . . . . . . . . . . . . .

**ADR- Report date:** day [ ] [ ] month [ ][ ] year [ 2 ][ 0 ][ 1 ][ ]

Report corroborated by SURNAME: ............................................

Designation of corroborator: ...........................................

Copy of this patient's ADR report should be lodged with:

i) THIS WARD's pharmacy (where pharmacy staff will check, and record, which of the suspected ADR-drugs were issued by THIS WARD's pharmacy)
ii) the patient's case-notes + make copy for ward’s surveillance-audit
iii) Senior doctor in-charge of THIS WARD, Dr.    .......................................................
iv) Uganda's National Pharmacovigilance Centre [P. O. Box 23096, Kampala, Uganda or email: [ndaug@nda.org.ug](mailto:ndaug@nda.org.ug) or Tel: 255665/347391]

Thank you for helping to improve medicines and patient safety, both in THIS WARD and nationally.

Signature of senior doctor in-charge of THIS WARD: .......................................................
Signature of Ronald Kiguba: .......................................................

Questions about this form may be addressed to Ronald Kiguba (mobile: 0712840683).
On a 4-weekly basis, THIS WARD, its pharmacy and senior doctor in-charge review all corroborated ADR-reports from THIS WARD.
The 4-weekly ADR-summaries for THIS WARD are available in THIS WARD's pharmacy: please consult them. THANK YOU because your reporting has contributed to the improvements in patient-safety information that the reports highlight.
