# Supplementary material for: Antibiotic‐associated suspected adverse drug reactions among hospitalized patients in Uganda: a prospective cohort study
Source: Pharmacol Res Perspect. 2017 Feb 17;5(2):e00298. doi: 10.1002/prp2.298 (PMC5368962; doi:10.1002/prp2.298)
Supplement: Supplementary file 2 — Table S1. (A) Incidence of hospital‐acquired antibiotic‐associated suspected ADRs per 100 Defined Daily Doses stratified by HIV‐status.(B) Incidence of hospital‐acquired antibiotic‐associated suspected ADRs per 100 patients at risk stratified by HIV‐status. [file PRP2-5-e00298-s002.doc]

**Table S1A: Incidence of hospital-acquired antibiotic-associated suspected ADRs per 100 Defined Daily Doses stratified by HIV-status**

| Drug Name | Number of patients on drug while in Hospital | | Number of patients with hospital-acquired ADRs | | Number of hospital-acquired ADRs | | Defined Daily Doses used in Hospital | | Number of hospital-acquired ADRs/100 Defined Daily Doses | | | |
| --- | --- | --- | --- | --- | --- | --- | --- | --- | --- | --- | --- | --- |
|  | HIV+ | HIV-/NK | HIV+ | HIV-/NK | HIV+ | HIV-/NK | HIV+ | HIV-/NK | HIV+ | 95% CI | HIV-/NK | 95% CI |
| Ceftriaxone | 147 | 251 | 20 | 44 | 28 | 66 | 147 | 251 | 19 | 13 -26 | 26 | 21-32 |
| Metronidazole | 74 | 172 | 9 | 19 | 11 | 33 | 110 | 199 | 10 | 5- 17 | 17 | 12-22 |
| Levofloxacin | 3 | 14 | 1 | 4 | 1 | 14 | 11 | 59 | 9 | 0- 51 | 24 | 14-37 |

**Table S1B: Incidence of hospital-acquired antibiotic-associated suspected ADRs per 100 patients at risk stratified by HIV-status**

| Drug Name | No. of patients on drug in Hospital | | No. of patients with hospital-acquired ADR | | Incidence of hospital-acquired ADRs (Percent) | | | |  |
| --- | --- | --- | --- | --- | --- | --- | --- | --- | --- |
|  | HIV+ | HIV-/NK | HIV+ | HIV-/NK | HIV+ | 95% CI - HIV+ | HIV-/NK | 95% CI - HIV-/NK | Overall |
| Ceftriaxone | 147 | 251 | 20 | 44 | 14 | 9 - 20 | 18 | 13 -23 | 16 |
| Metronidazole | 74 | 172 | 9 | 19 | 12 | 6 - 23 | 11 | 7- 17 | 11 |
| Levofloxacin | 3 | 14 | 1 | 4 | 33 | 1-100 | 29 | 8- 73 | 29 |

ADRs: Adverse Drug Reactions; NK: Not Known; CI: Confidence Interval; HIV+: HIV-positive serostatus; HIV-: HIV-negative serostatus
