# Supplementary material for: Antibiotic‐associated suspected adverse drug reactions among hospitalized patients in Uganda: a prospective cohort study
Source: Pharmacol Res Perspect. 2017 Feb 17;5(2):e00298. doi: 10.1002/prp2.298 (PMC5368962; doi:10.1002/prp2.298)
Supplement: Supplementary file 3 — Table S2. Frequencies of individual antibiotic‐associated ADRs in the Probable or Definite vs. Possible ADR causality categories, Uganda, 2014. [file PRP2-5-e00298-s003.doc]

**Table S2: Frequencies of individual antibiotic-associated ADRs in the Probable or Definite vs. Possible ADR causality categories, Uganda, 2014**

| No. | Possible ADRs | Frequency | % |
| --- | --- | --- | --- |
| 1 | Vomiting | 33 | 16 |
| 2 | Loss of appetite | 25 | 12 |
| 3 | Dizziness | 24 | 12 |
| 4 | Fever | 19 | 9 |
| 5 | Headache | 15 | 7 |
| 6 | Diarrhoea | 15 | 7 |
| 7 | Nausea | 14 | 7 |
| 8 | Itching & Skin Rash | 9 | 4 |
| 9 | Peripheral neuropathy | 8 | 4 |
| 10 | Abdominal pain | 8 | 4 |
| 11 | Anaemia/skin parlour | 5 | 2 |
| 12 | Flatulence/abdominal discomfort | 4 | 2 |
| 13 | Jaundice | 4 | 2 |
| 14 | Constipation | 4 | 2 |
| 15 | Cough & shortness of breath | 3 | 1 |
| 16 | Palpitations | 2 | 1 |
| 17 | General Body Weakness | 2 | 1 |
| 18 | Tachycardia | 2 | 1 |
| 19 | Dyspepsia | 1 | 0 |
| 20 | DIB | 1 | 0 |
| 21 | Blurred vision | 1 | 0 |
| 22 | Joint pain | 1 | 0 |
| 23 | Convulsion | 1 | 0 |
| 24 | Oral sores | 1 | 0 |
| 25 | Dysuria | 1 | 0 |
| **TOTAL** | | **203** | **100** |

| No. | Probable or Definite ADRs | Frequency | % |
| --- | --- | --- | --- |
| 1 | Vomiting | 13 | 20 |
| 2 | Peripheral neuropathy | 6 | 9 |
| 3 | Itching & Skin Rash | 6 | 9 |
| 4 | Diarrhoea | 6 | 9 |
| 5 | Abdominal pain | 5 | 8 |
| 6 | Headache | 5 | 8 |
| 7 | Dizziness | 4 | 6 |
| 8 | Nausea | 3 | 5 |
| 9 | Fever | 3 | 5 |
| 10 | General Body Weakness | 3 | 5 |
| 11 | Anaemia | 3 | 5 |
| 12 | Jaundice | 2 | 3 |
| 13 | Hypertension | 1 | 2 |
| 14 | Loose stool | 1 | 2 |
| 15 | Loss of appetite | 1 | 2 |
| 16 | Constipation | 1 | 2 |
| 17 | Haemorrhoids | 1 | 2 |
| 18 | DIB with shortness of breath | 1 | 2 |
| 19 | Pedal Oedema | 1 | 2 |
| TOTAL | | **66** | **100** |

DIB: Difficulty in Breathing
