# Supplementary material for: Antibiotic‐associated suspected adverse drug reactions among hospitalized patients in Uganda: a prospective cohort study
Source: Pharmacol Res Perspect. 2017 Feb 17;5(2):e00298. doi: 10.1002/prp2.298 (PMC5368962; doi:10.1002/prp2.298)
Supplement: Supplementary file 4 — Table S3. Serious antibiotic‐associated suspected adverse drug reactions, with preventability, experienced by hospitalized patients, Uganda, 2014. [file PRP2-5-e00298-s004.doc]

| **Table S3: Serious antibiotic-associated suspected adverse drug reactions, with preventability, experienced by hospitalized patients , Uganda, 2014** | | | | | | | | |
| --- | --- | --- | --- | --- | --- | --- | --- | --- |
| **Adverse Drug Reaction** | **Drug** | **Severity** | **Causality** | **Rarity** | **HIV-status** | **Community or Hospital- acquired** | **Grade of Seriousness** | **Preventability** |
| FEVER | INH, RIFAMPICIN, PYRAZINAMIDE | Moderate | Possible | No | Negative | Community-acquired | Required intervention to prevent damage | Probably Preventable |
| DIZZINESS | CEFTRIAXONE | Severe | Possible | No | Negative | Hospital-acquired | Required intervention to prevent damage | Probably Preventable |
| FEVER | CEFTRIAXONE | Severe | Possible | No | Negative | Hospital-acquired | Required intervention to prevent damage | Probably Preventable |
| VOMITING | CEFTRIAXONE, TRAMADOL | Severe | Possible | No | Negative | Hospital-acquired | Required intervention to prevent damage | Probably Preventable |
| FEVER | CEFTRIAXONE | Severe | Possible | No | Negative | Hospital-acquired | Required intervention to prevent damage | Probably Preventable |
| DIZZINESS | CEFTRIAXONE, METRONIDAZOLE | Moderate | Possible | No | Negative | Hospital-acquired | Required intervention to prevent damage | Not Preventable |
| ANAEMIA | UNKNOWN HERBAL, CEFTRIAXONE, CAPTOPRIL | Moderate | Possible | No | Negative | Hospital-acquired | Required intervention to prevent damage | Not Preventable |
| PERIPHERAL NEUROPATHY | LEVOFLOXACIN | Moderate | Possible | No | Negative | Hospital-acquired | Caused or prolonged Hosp | Probably Preventable |
| TACHYCARDIA | CIPROFLOXACIN | Moderate | Possible | No | Negative | Hospital-acquired | Required intervention to prevent damage | Probably Preventable |
| DIZZINESS | METRONIDAZOLE, AMOXICILLIN | Severe | Possible | NK | Negative | Hospital-acquired | Caused or prolonged Hosp | Probably Preventable |
| CONVULSIONS - GTC (2 EPISODES) | METRONIDAZOLE | Severe | Possible | No | Negative | Hospital-acquired | Required intervention to prevent damage | Probably Preventable |
| WORSENED JAUNDICE | CIPROFLOXACIN, CEFTRIAXONE, METRONIDAZOLE | Moderate | Possible | No | Negative | Hospital-acquired | Other medically significant condition | Not Preventable |
| VOMITING | CEFTRIAXONE | Severe | Possible | No | Negative | Hospital-acquired | Other medically significant condition | Probably Preventable |
| ORAL SORES | CEFTRIAXONE | Moderate | Possible | No | Negative | Hospital-acquired | Other medically significant condition | Not Preventable |
| HYPERTENSION | CIPROFLOXACIN | Severe | Probable | No | Negative | Hospital-acquired | Required intervention to prevent damage | Not Preventable |
| PARAESTHESIA | CEFTRIAXONE | Moderate | Probable | NK | Negative | Hospital-acquired | Other medically significant condition | Not Preventable |
| ITCHING SKIN - MULTIFORME RASH | DICLOFENAC, METRONIDAZOLE | Moderate | Probable | No | Negative | Hospital-acquired | Required intervention to prevent damage | Not Preventable |
| PARAESTHESIAS (PERIPHERAL NEUROPATH | HRZE | Severe | Possible | No | Unknown | Community-acquired | Required intervention to prevent damage | Not Preventable |
| HIGH GRADE FEVER WITH CHILLS AND RIGO | HRZE | Severe | Possible | No | Unknown | Community-acquired | Caused or prolonged Hosp | Probably Preventable |
| BLURRED VISION | METRONIDAZOLE | Mild | Possible | Yes | Unknown | Hospital-acquired | Other medically significant condition | Probably Preventable |
| VOMITING (3 EPISODES) | CEFTRIAXONE, TRAMADOL | Mild | Possible | Yes | Unknown | Hospital-acquired | Other medically significant condition | Probably Preventable |
| LOSS OF APPETITE | METRONIDAZOLE | Moderate | Possible | No | Unknown | Hospital-acquired | Caused or prolonged Hosp | Not Preventable |
| DECREASED URINE OUTPUT | CEFTRIAXONE | Severe | Probable | Yes | Unknown | Hospital-acquired | Other medically significant condition | Not Preventable |
|  |  |  |  |  |  |  |  |  |
| LOSS OF APPETITE | CTX & METRONIDAZOLE | Moderate | Possible | No | Positive | Community-acquired | Required intervention to prevent damage | Probably Preventable |
| PARAESTHESIAS | 3TC, CTX | Moderate | Possible | No | Positive | Community-acquired | Other medically significant condition | Probably Preventable |
| FEVER | AZT/3TC/EFV, CTX | Severe | Possible | No | Positive | Community-acquired | Required intervention to prevent damage | Probably Preventable |
| NUMBNESS OF BOTH LOWER LIMBS | HE, 3TC, CTX | Moderate | Possible | No | Positive | Community-acquired | Required intervention to prevent damage | Probably Preventable |
| DIFFICULTY IN BREATHING | RIFAMPICIN, TDF/3TC, CTX | Severe | Possible | No | Positive | Community-acquired | Required intervention to prevent damage | Probably Preventable |
| PRODUCTIVE COUGH | CTX, TDF/3TC | Severe | Possible | No | Positive | Community-acquired | Required intervention to prevent damage | Probably Preventable |
| SEVERE PALLOR OF MUCUS MEMBRANES | HRZE, TDF/3TC/NVP, CTX | Severe | Possible | No | Positive | Community-acquired | Required intervention to prevent damage | Probably Preventable |
| DIZZINESS | HRZE, TDF/3TC, CTX | Severe | Possible | No | Positive | Community-acquired | Caused or prolonged Hosp | Probably Preventable |
| JAUNDICE | HRZE, TDF/3TC, CTX | Moderate | Possible | No | Positive | Community-acquired | Required intervention to prevent damage | Probably Preventable |
| DIZZINESS | COARTEM, DAPSONE, FLUCONAZOLE | Moderate | Possible | No | Positive | Community-acquired | Required intervention to prevent damage | Probably Preventable |
| PARAPARESIS | INH | Moderate | Possible | No | Positive | Community-acquired | Caused or prolonged Hosp | Probably Preventable |
| JAUNDICE | INH | Life-threate | Possible | No | Positive | Community-acquired | Caused death | Not Preventable |
| GENERALIZED MACULO-PAPULAR RASH | CTX, ARVS (TDF/3TC/EFV) | Moderate | Possible | No | Positive | Community-acquired | Caused or prolonged Hosp | Not Preventable |
| FEVER 38.3C | ACICLOVIR, CEFTRIAXONE | Severe | Possible | Yes | Positive | Community-acquired | Caused or prolonged Hosp | Not Preventable |
| WORSENED PALLOR | TDF/3TC, CTX | Severe | Possible | No | Positive | Community-acquired | Required intervention to prevent damage | Probably Preventable |
| DIARRHOEA | TDF/3TC/EFV, CTX | Moderate | Possible | No | Positive | Community-acquired | Caused or prolonged Hosp | Probably Preventable |
| SEVERE ANAEMIA 3.4G/DL | TDF/3TC, CTX | Severe | Possible | No | Positive | Community-acquired | Caused or prolonged Hosp | Probably Preventable |
| HEADACHE | CTX | Moderate | Possible | Yes | Positive | Community-acquired | Caused or prolonged Hosp | Probably Preventable |
| VOMITING | HRZE | Severe | Possible | No | Positive | Community-acquired | Caused or prolonged Hosp | Probably Preventable |
| ANAEMIA | CTX | Moderate | Possible | No | Positive | Community-acquired | Caused or prolonged Hosp | Probably Preventable |

| **Adverse Drug Reaction** | **Drug** | **Severity** | **Causality** | **Rarity** | **HIV-status** | **Community or Hospital- acquired** | **Grade of Seriousness** | **Preventability** |
| --- | --- | --- | --- | --- | --- | --- | --- | --- |
| ABD DISCOMFORT/DISTENTION | HRZE, AZT | Moderate | Possible | No | Positive | Community-acquired | Other medically significant condition | Not Preventable |
| PARAESTHESIAS | CTX, TDF/3TC/LPV/RTV | Moderate | Possible | No | Positive | Community-acquired | Required intervention to prevent damage | Probably Preventable |
| PERSISTENT DIARRHOEA | 3TC/TDF/EFV, CTX | Severe | Possible | No | Positive | Community-acquired | Caused or prolonged Hosp | Not Preventable |
| VOMITING | 3TC/TDF/EFV, CTX | Severe | Possible | No | Positive | Community-acquired | Required intervention to prevent damage | Probably Preventable |
| VOMITING | CEFTRIAXONE | Severe | Possible | No | Positive | Community-acquired | Caused or prolonged Hosp | Not Preventable |
| JOINT PAIN | 3TC/TDF, CTX | Severe | Possible | No | Positive | Community-acquired | Required intervention to prevent damage | Not Preventable |
| PERIPHERAL NEUROPATHY | AZT, 3TC, INH | Moderate | Possible | No | Positive | Community-acquired | Other medically significant condition | Probably Preventable |
| DEEP JAUNDICE | RHZ, AZT/3TC | Severe | Possible | No | Positive | Community-acquired | Required intervention to prevent damage | Probably Preventable |
| COUGH | TDF/3TC, CTX | Severe | Possible | No | Positive | Community-acquired | Required intervention to prevent damage | Probably Preventable |
| DRY COUGH WITH SHORTNESS OF BREATH | CTX | Severe | Possible | Yes | Positive | Community-acquired | Required intervention to prevent damage | Probably Preventable |
| DIZZINESS | METRONIDAZOLE | Severe | Possible | Yes | Positive | Community-acquired | Required intervention to prevent damage | Not Preventable |
| DIZZINESS | TDF/3TC, RHZE | Moderate | Probable | No | Positive | Community-acquired | Required intervention to prevent damage | Probably Preventable |
| LOSS OF APPETITE | 3TC, TDF, RIFAMPICIN, PYRAZINAMIDE | Moderate | Probable | No | Positive | Community-acquired | Required intervention to prevent damage | Probably Preventable |
| GENERALIZED BODY WEAKNESS | TDF/3TC, RIFAMPICIN | Moderate | Probable | No | Positive | Community-acquired | Caused or prolonged Hosp | Probably Preventable |
| VOMITING | HRZE | Moderate | Probable | No | Positive | Community-acquired | Required intervention to prevent damage | Probably Preventable |
| CONSTIPATION | INH | Moderate | Probable | No | Positive | Community-acquired | Required intervention to prevent damage | Probably Preventable |
| HEADACHE | INH, RIFAMPICIN | Moderate | Probable | No | Positive | Community-acquired | Required intervention to prevent damage | Probably Preventable |
| PRURITUS | TDF/EFV, METRONIDAZOLE, INH, ETHAMBUTOL | Severe | Probable | No | Positive | Community-acquired | Caused or prolonged Hosp | Not Preventable |
| ITCHY RASH WITH NUMBNESS OF LOWER S | HE, CARVEDILOL | Moderate | Probable | No | Positive | Community-acquired | Caused disability | Not Preventable |
| JAUNDICE | CTX | Moderate | Probable | Yes | Positive | Community-acquired | Required intervention to prevent damage | Not Preventable |
| VOMITING | ERYTHROMYCIN, CEFTRIAXONE | Severe | Probable | No | Positive | Community-acquired | Required intervention to prevent damage | Probably Preventable |
| PERIPHERAL NEUROPATHY | 3TC, METRONIDAZOLE | Moderate | Probable | No | Positive | Community-acquired | Caused or prolonged Hosp | Not Preventable |
| DIB WITH SHORTNESS OF BREATH | RIFAMPCIN | Severe | Probable | No | Positive | Community-acquired | Life-threatening | Probably Preventable |
| ANAEMIA | CTX, TDF/3TC/LPV/RTV | Moderate | Probable | No | Positive | Community-acquired | Caused or prolonged Hosp | Not Preventable |
| PEDAL OEDEMA | RIFAMPICIN | Severe | Probable | No | Positive | Community-acquired | Required intervention to prevent damage | Not Preventable |
| PARAESTHESIA | HRZE | Mild | Probable | No | Positive | Community-acquired | Caused or prolonged Hosp | Not Preventable |
| DIZZINESS | CEFTRIAXONE,METRONIDAZOLE,DICLOFENAC | Moderate | Possible | No | Positive | Hospital-acquired | Required intervention to prevent damage | Probably Preventable |
| VOMITING | CEFTRIAXONE, METRONIDAZOLE, BLOOD, DICLOFENAC | Severe | Possible | No | Positive | Hospital-acquired | Required intervention to prevent damage | Probably Preventable |
| FEVER | CTX, TDF/3TC/EFV | Moderate | Possible | No | Positive | Hospital-acquired | Required intervention to prevent damage | Probably Preventable |
| FEVER | CEFTRIAXONE | Moderate | Possible | No | Positive | Hospital-acquired | Required intervention to prevent damage | Probably Preventable |
| VOMITING | CEFTRIAXONE, TDF/3TC/EFV | Severe | Possible | No | Positive | Hospital-acquired | Caused or prolonged Hosp | Not Preventable |
| DIZZINESS | CEFTRIAXONE, METOCLOPRAMIDE | Moderate | Possible | No | Positive | Hospital-acquired | Caused or prolonged Hosp | Not Preventable |
| VOMITING | CEFTRIAXONE | Moderate | Possible | No | Positive | Hospital-acquired | Caused or prolonged Hosp | Probably Preventable |
| FEVER 39.6C | CEFTRIAXONE | Severe | Possible | No | Positive | Hospital-acquired | Caused or prolonged Hosp | Not Preventable |
| VOMITING | FLUCONAZOLE, ACICLOVIR, CEFTRIAXONE | Moderate | Possible | No | Positive | Hospital-acquired | Caused or prolonged Hosp | Probably Preventable |
| ANOREXIA | CTX | Moderate | Possible | No | Positive | Hospital-acquired | Required intervention to prevent damage | Probably Preventable |
| DIARRHOEA | CEFTRIAXONE | Moderate | Possible | No | Positive | Hospital-acquired | Required intervention to prevent damage | Not Preventable |
| SEVERE ABD PAIN | CIPROFLOXACIN | Severe | Possible | No | Positive | Hospital-acquired | Required intervention to prevent damage | Not Preventable |
| ANOREXIA | 3TC/TDF, CTX | Moderate | Possible | No | Positive | Hospital-acquired | Required intervention to prevent damage | Not Preventable |
| HIGH GRADE FEVER - 39.2 C | CEFTRIAXONE | Severe | Possible | No | Positive | Hospital-acquired | Required intervention to prevent damage | Not Preventable |
| FEVER 38.5C | CEFTRIAXONE | Moderate | Probable | No | Positive | Hospital-acquired | Required intervention to prevent damage | Not Preventable |
| FEVER 38 C | CEFTRIAXONE, DUOVIR-N, INH, PYRAZINAMIDE | Mild | Probable | No | Positive | Hospital-acquired | Required intervention to prevent damage | Not Preventable |
| VOMITING | CEFTRIAXONE | Moderate | Probable | No | Positive | Hospital-acquired | Required intervention to prevent damage | Not Preventable |
| CTX: Co-trimoxazole; INH: Isoniazid; HE: Isoniazid/Ethambutol; RHZ: Rifampicin/Isoniazid/Pyrazinamide; HRZE: Isoniazid/rifampicin/pyrazinamide/ethambutol; AZT: Zidovudine; TDF: Tenofovir; 3TC: lamivudine; NVP: nevirapine; EFV: Efavirenz; LPV: Lopinavir; RTV: Ritonavir; ARVS: Antiretrovirals; DUOVIR-N: Zidovudine/lamivudine/nevirapine; NK: Incidence unknown | | | | | | | | |
