# Supplementary material for: Antibiotic‐associated suspected adverse drug reactions among hospitalized patients in Uganda: a prospective cohort study
Source: Pharmacol Res Perspect. 2017 Feb 17;5(2):e00298. doi: 10.1002/prp2.298 (PMC5368962; doi:10.1002/prp2.298)
Supplement: Supplementary file 5 — Table S4. List of 24 rare antibiotic‐associated suspected adverse drug reactions experienced by hospitalized patients, Uganda, 2014. [file PRP2-5-e00298-s005.doc]

**Table S4: List of 24 rare antibiotic-associated suspected adverse drug reactions experienced by hospitalized patients, Uganda, 2014**

| **adr** | **drug** | **severity** | **causality** | **preventability** | **serious** | **Community/Hospital-acquired** |
| --- | --- | --- | --- | --- | --- | --- |
| DRY COUGH WITH SHORTNESS OF BREATH | CO-TRIMOXAZOLE | Severe | Possible | Probably Preventable | Yes | Community-acquired |
| JAUNDICE | CO-TRIMOXAZOLE | Moderate | Probable | Not Preventable | Yes | Community-acquired |
| ANOREXIA | CO-TRIMOXAZOLE | Moderate | Possible | Probably Preventable | Yes | Community-acquired |
| ANAEMIA | CO-TRIMOXAZOLE | Moderate | Possible | Probably Preventable | Yes | Community-acquired |
| DECREASED APPETITE | CO-TRIMOXAZOLE | Mild | Possible | Probably Preventable | No | Community-acquired |
| ANOREXIA | CO-TRIMOXAZOLE | Mild | Possible | Probably Preventable | No | Community-acquired |
| FEVER | CO-TRIMOXAZOLE, FEFOL | Moderate | Possible | Not Preventable | No | Community-acquired |
| ABDOMINAL PAIN | CEFTRIAXONE & CO-TRIMOXAZOLE | Mild | Possible | Probably Preventable | No | Community-acquired |
| CONSTIPATION | METRONIDAZOLE | Moderate | Possible | Probably Preventable | No | Community-acquired |
| HARD STOOL | METRONIDAZOLE | Moderate | Possible | Probably Preventable | No | Community-acquired |
| FEVER | METRONIDAZOLE | Mild | Possible | Not Preventable | No | Community-acquired |
| DIZZINESS | METRONIDAZOLE | Severe | Possible | Not Preventable | Yes | Community-acquired |
|  |  |  |  |  |  |  |
| CONVULSIONS - GTC (2 EPISODES) | METRONIDAZOLE | Severe | Possible | Probably Preventable | Yes | Hospital-acquired |
| CONSTIPATION | METRONIDAZOLE | Moderate | Possible | Probably Preventable | No | Hospital-acquired |
| DIZZINESS | METRONIDAZOLE | Mild | Possible | Probably Preventable | No | Hospital-acquired |
| DIZZINESS | METRONIDAZOLE | Mild | Possible | Probably Preventable | No | Hospital-acquired |
| BLURRED VISION | METRONIDAZOLE | Mild | Possible | Probably Preventable | Yes | Hospital-acquired |
| FACIAL ITCHY SKIN RASH | METRONIDAZOLE | Mild | Possible | Probably Preventable | No | Hospital-acquired |
| HEADACHE | METRONIDAZOLE | Mild | Possible | Probably Preventable | No | Hospital-acquired |
| DIZZINESS | METRONIDAZOLE | Mild | Possible | Probably Preventable | No | Hospital-acquired |
| PERIPHERAL NEUROPATHY | LEVOFLOXACIN | Moderate | Possible | Probably Preventable | Yes | Hospital-acquired |
| DECREASED URINE OUTPUT | CEFTRIAXONE | Severe | Probable | Not Preventable | Yes | Hospital-acquired |
| DECREASED APPETITE | CEFTRIAXONE | Moderate | Possible | Not Preventable | No | Hospital-acquired |
| LOSS OF APPETITE | CEFTRIAXONE | Moderate | Possible | Probably Preventable | No | Hospital-acquired |
